# Application of a Screen-Printed Sensor Modified with Carbon Nanofibers for the Voltammetric Analysis of an Anticancer Disubstituted Fused Triazinone

**DOI:** 10.3390/ijms23052429

**Published:** 2022-02-23

**Authors:** Jędrzej Kozak, Katarzyna Tyszczuk-Rotko, Ilona Sadok, Krzysztof Sztanke, Małgorzata Sztanke

**Affiliations:** 1 Faculty of Chemistry, Institute of Chemical Sciences, Maria Curie-Skłodowska University in Lublin, 20-031 Lublin, Poland; jedrekkozak@onet.pl (J.K.); katarzyna.tyszczuk-rotko@mail.umcs.pl (K.T.-R.); 2 Laboratory of Separation and Spectroscopic Method Applications, Centre for Interdisciplinary Research, Faculty of Science and Health, The John Paul II Catholic University of Lublin, 20-708 Lublin, Poland; ilona.sadok@kul.pl; 3 Laboratory of Bioorganic Synthesis and Analysis, Chair and Department of Medical Chemistry, Medical University of Lublin, 4A Chodźki Street, 20-093 Lublin, Poland; 4 Chair and Department of Medical Chemistry, Medical University of Lublin, 4A Chodźki Street, 20-093 Lublin, Poland; malgorzata.sztanke@umlub.pl

**Keywords:** disubstituted fused triazinone, anticancer agent candidate, square-wave adsorptive stripping voltammetry, screen-printed sensor, carbon nanofibers, flow system

## Abstract

In this paper, we propose the first analytical procedure—using a screen-printed carbon electrode modified with carbon nanofibers (SPCE/CNFs)—for the detection and quantitative determination of an electroactive disubstituted fused triazinone, namely 4-Cl-PIMT, which is a promising anticancer drug candidate. The electrochemical performances of the sensor were investigated by cyclic voltammetry (CV), electrochemical impedance spectroscopy (EIS), and square-wave adsorptive stripping voltammetry (SWAdSV). The presence of carbon nanofibers on the sensor surface caused a decrease in charge-transfer resistance and an increase in the active surface compared to the bare SPCE. Under the optimised experimental conditions, the proposed voltammetric procedure possesses a good linear response for the determination of 4-Cl-PIMT in the two linear ranges of 0.5–10 nM and 10–100 nM. The low limits of detection and quantification were calculated at 0.099 and 0.33 nM, respectively. In addition, the sensor displays high reproducibility and repeatability, as well as good selectivity. The selectivity was improved through the use of a flow system and a short accumulation time. The SWAdSV procedure with SPCE/CNFs was applied to determine 4-Cl-PIMT in human serum samples. The SWAdSV results were compared to those obtained by the ultra-high-performance liquid chromatography coupled with electrospray ionization/single-quadrupole mass spectrometry (UHPLC-ESI-MS) method.

## 1. Introduction

**Figure 1:**
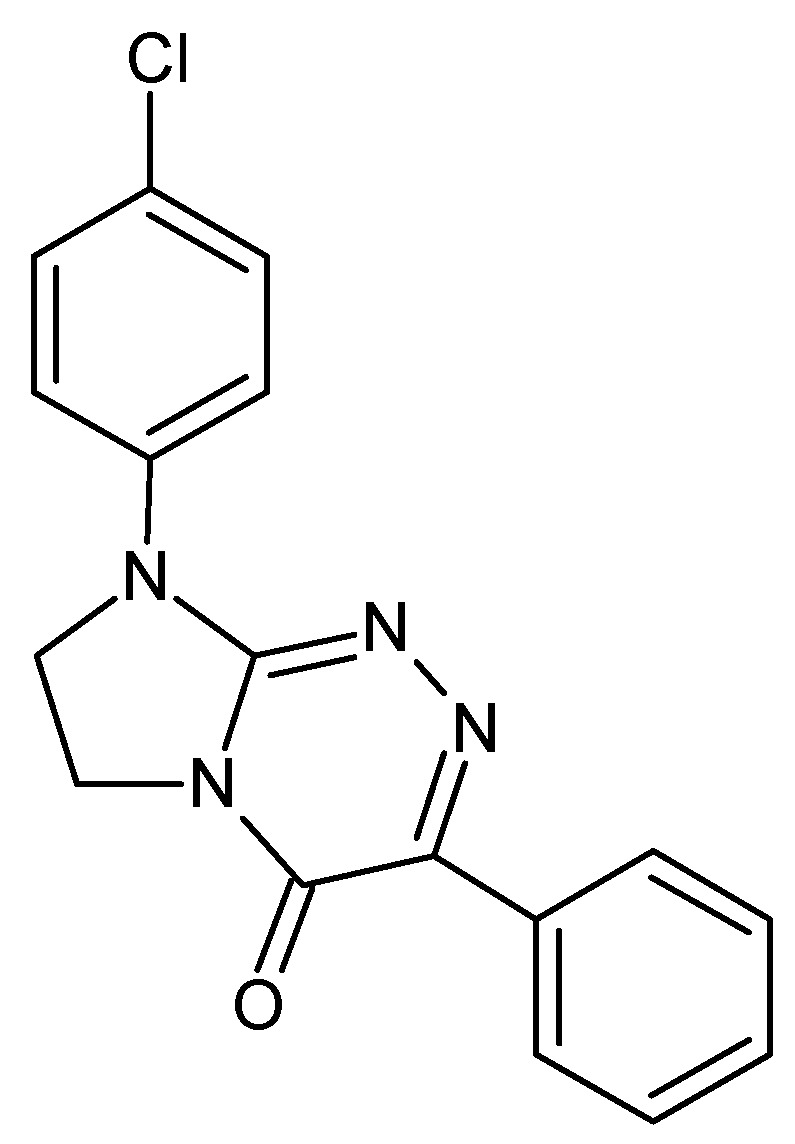
8-(4-Chlorophenyl)-3-phenyl-7,8-dihydroimidazo[2,1-*c*][1,2,4]triazin-4(6*H*)-one (4-Cl-PIMT)—an electroactive molecule used in this study.

Among pharmaceutically important disubstituted fused triazinones, 4-Cl-PIMT (i.e., 8-(4-chlorophenyl)-3-phenyl-7,8-dihydroimidazo[2,1-*c*][1,2,4]triazin-4(6*H*)-one; Figure 1), with a fully defined molecular structure, is the most promising anticancer drug candidate. This innovative nucleobase-like molecule revealed significant, concentration-dependent antiproliferative effects in human peripheral blood myeloma cells, indicating a possible usefulness in the treatment of such haematological malignancies. Furthermore, this compound proved to have the strongest antimigratory property in human tumour cells of the cervix among all disubstituted fused triazinones, suggesting the highest antimetastatic potential and, consequently, an applicability in the prevention of metastasis [1]. It is worth noting that 4-Cl-PIMT, as a promising candidate for an anticancer agent, was found to be completely nontoxic to normal human skin fibroblast [1] and African green monkey kidney (data not published) cells and low in toxicity for mice (LD_50_ > 2000 mg kg^−1^, i.p.) [2]. This compound was predicted (using OSIRIS Property Explorer: http://www.organic-chemistry.org/prog/peo/) (accessed on 7 February 2022) to have no adverse side effects, such as mutagenicity, tumorigenicity, irritating and reproductive effects, due to the lack of “genotoxicophore” fragments in its structure. Based on the highly significant QSARs between the experimentally determined lipophilicity parameters (on columns imitating biosystems) and pharmacokinetic parameters, as well as the reliable and predictive QSAR models developed for assessing the penetration of the blood–brain barrier, this small molecule could be expected to have good bioavailability and high permeability through biological barriers [2,3,4]. The ability to penetrate the blood–brain barrier was also confirmed by its effect on the central nervous system in mice [2]. The above features indicate that this anticancer fused triazinone can be qualified as a promising candidate for further pharmacological in vivo studies.

Chromatography and spectroscopy are among the popular methods used in the analysis of organic compounds, especially pharmaceuticals. However, these techniques are time-consuming, labour-intensive, and generate a high consumption of reagents. In addition, they require expensive equipment, and due to their complexity, highly qualified analysts are required [5,6]. In contrast to the above-mentioned methods, electrochemical methods, which include voltammetry, are characterised by a relatively low cost and simplicity of operations, as well as high stability and sensitivity [5,7]. Electrochemical analysis is practically free of organic solvents, is fast, and can be used on a large scale, thanks to portable, miniaturised tools [6,8,9]. Electrochemical methods, including voltammetry, use electrodes as a conversion element, the surface of which interacts with the analyte and converts the information obtained into a signal that can be measured and identified by means of electrochemical analysis control devices [10]. The most frequently used method in quantitative analysis is voltammetry coupled with pulse waveform and the accumulation of analyte on the electrode surface, e.g., square-wave adsorptive stripping voltammetry (SWAdSV). Techniques of this kind are considered a tool to obtain the very low limits of detection (LODs) and quantification (LOQs) [11].

A whole range of working electrodes is used in voltammetry, e.g., mercury electrodes, glassy carbon electrodes, carbon paste electrodes, or boron-doped diamond electrodes [12]. Since the initial development of screen-printed carbon electrodes (SPCEs) in the 1980s, these sensors have found applications in biomedical, environmental, and industrial analyses. Unlike individual electrodes in traditional electrochemical analysis, all SPCEs are printed and integrated on an inert plastic or ceramic substrate, where a carbon ink and silver pseudo-electrodes usually serve as working, counter, and reference electrodes [13,14]. Screen-printing techniques offer simple means of possible mass production of disposable, stable, and low-cost screen-printed electrodes to be used in on-site analyses [15,16]. Screen-printed electrodes are often employed in analytical applications due to their specific characteristics, including small dimensions and determination limits, quick response duration, reproducibility, ability to operate at room temperature, and low background current [5,17,18]. Additional advantages of screen-printed electrodes are diversification of the selection of electrode materials, portability, reliability for detecting different substances, and an ease of surface modification for various uses [19].

Often, both conventional and screen-printed electrodes require surface modification for improved selectivity and sensitivity in determining the analyte. The modifier applied to the electrode increases its active surface and improves electron transfer. Among the currently popular modifiers, various types of nanoparticles and nanomaterials are used, including carbon nanomaterials [12,20]. Nanomaterials are chemicals or materials that are produced and used on a very small scale. Nanomaterials are being developed to exhibit new properties compared to the same material without nanoscale features [21]. Carbon-based nanomaterials have attracted much attention due to their large surface area, interconnecting network, low electrical resistance, and excellent electrical conductivity, as well as good chemical and physical stability toward electrochemical purposes [22]. Among carbon nanomaterials, we can distinguish carbon nanofibers (CNFs); carbon nanohorns (CNHs); and carbon nanotubes, including single-walled (SWCNTs), double-walled (DWCNTs) and even multiwalled (MWCNTs) nanotubes [23,24,25]. Carbon nanofibers (CNFs) are graphitic materials with a high surface area-to-volume ratio and excellent mechanical strength, flexibility, chemical stability, and biocompatibility [26].

Despite the above-mentioned facts, no analytical procedure has been described in the literature to date for the quantitative determination of an anticancer fused triazinone (4-Cl-PIMT). On the other hand, this promising small molecule is the subject of our current electrochemical studies due to the attendance (in its structure) of an electroactive azomethine moiety of the ketimine-type, which may be the most susceptible to electrochemical reduction under experimental conditions. The purpose of the present study is to develop and optimise the first analytical procedure—using the most suitable screen-printed sensor coupled with a flow system—which would enable the detection and subsequent quantitative determination of this potential anticancer drug in both solution and enriched serum samples.

## 2. Results and Discussion

### 2.1. Initial Studies

**Figure 2:**
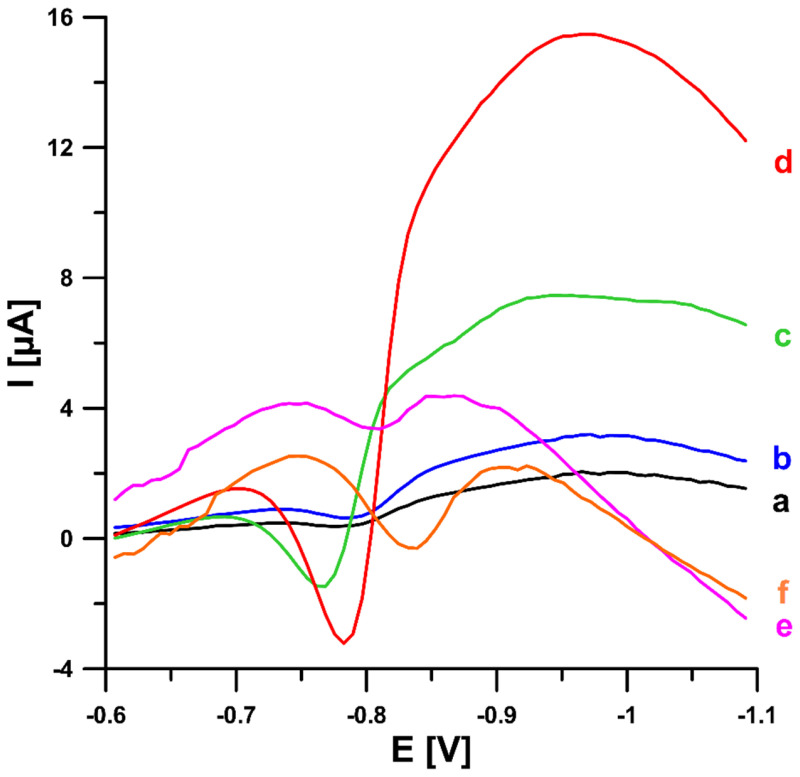
SW voltammograms of 0.05 (a, c, and e) and 0.1 µM (b, d, and f) 4-Cl-PIMT in 0.1 M H_2_SO_4_ solution at the screen-printed carbon electrode (SPCE) (a and b), screen-printed carbon electrode modified with carbon nanofibers (SPCE/CNFs) (c and d), and screen-printed carbon electrode modified with multiwalled carbon nanotubes (SPCE/MWCNTs) (e and f). SWV parameters: open-circuit potential, t of 45 s, initial E of −0.2 V, final E of −1.1 V, f of 50 Hz, E_SW_ of 50 mV, and ΔE of 7 mV.

In the initial stage of the experiments, we compared the voltammetric response of the title compound at the commercially available unmodified screen-printed carbon electrode (SPCE), screen-printed carbon electrode modified with carbon nanofibers (SPCE/CNFs), and screen-printed carbon electrode modified with multiwalled carbon nanotubes (SPCE/MWCNTs). SWV curves of 0.05 and 0.1 µM 4-Cl-PIMT were recorded at all electrodes after 45 s of solution stirring (Figure 2). The analytical signals obtained at the SPCE/CNFs were significantly improved compared to those obtained at the SPCE and the SPCE/MWCNTs. Therefore, the commercially available screen-printed carbon electrode modified with carbon nanofibers was selected for further study.

**Figure 3:**
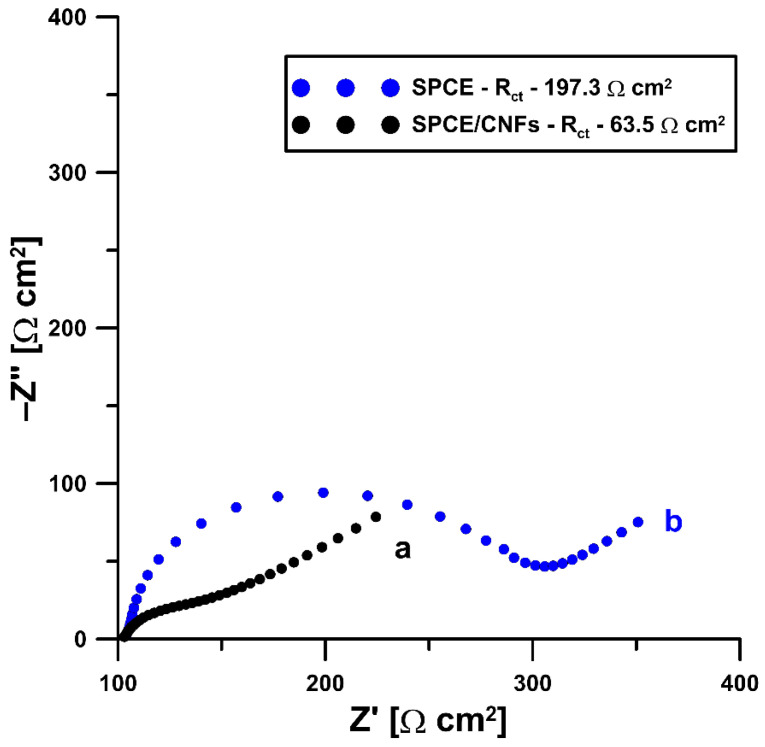
Nyquist plots of SPCE/CNFs (a) and SPCE (b).

To explain the advisability of modification of the electrode surface by carbon nanofibers, the commercially available, unmodified screen-printed carbon electrode (SPCE) and screen-printed carbon electrode modified with carbon nanofibers (SPCE/CNFs) were examined using the electrochemical impedance spectroscopy (EIS) method. Impedance spectra (Nyquist plots) were recorded in the frequency range from 20.0 kHz to 1.0 Hz at a potential of 0.2 V from a solution of 0.1 M KCl containing 5.0 mM K_3_[Fe(CN)_6_]. The obtained curves (Figure 3) clearly show that the presence of carbon nanofibers on the surface of the SPCE (black curve) causes a decrease in the charge-transfer resistance (R_ct_) compared to the bare SPCE (blue curve) (63.5 vs. 197.3 Ω cm^2^). Moreover, a better electrochemical performance of the SPCE/CNFs compared to the SPCE is observed as a result of the increase in its active surface due to modification (0.0809 ± 0.0014 vs. 0.061 ± 0.00058 cm^2^ (*n* = 3), respectively) [27,28,29].

### 2.2. Effect of Type and pH of Supporting Electrolytes

**Figure 4:**
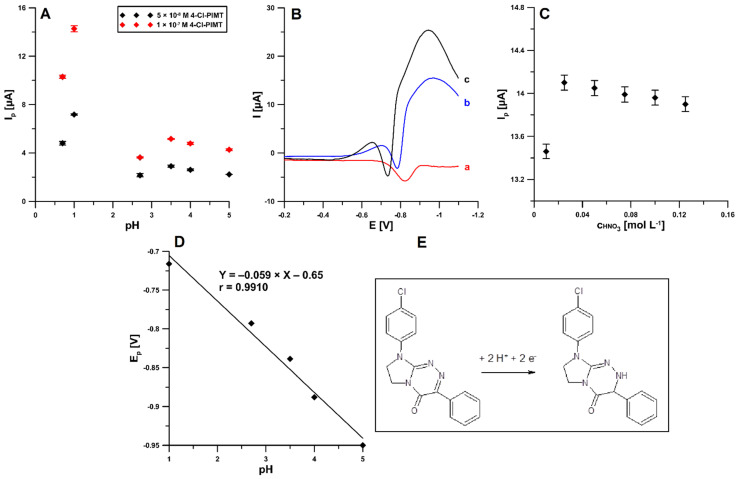
(**A**) Influence of the type of supporting electrolytes (0.1 M H_2_SO_4_, HNO_3_, CH_3_COOH and acetate buffers with pH values of 3.5 ± 0.1, 4.0 ± 0.1, and 5.0 ± 0.1) on the 4-Cl-PIMT peak current. The mean values of I_p_ are given with the standard deviation for *n* = 3. (**B**) SWV curves recorded in 0.1 M solutions of acetic acid (a), sulfuric acid (b), and nitric acid (c) containing 0.1 µM 4-Cl-PIMT. (**C**) Influence of the concentration of nitric acid on the peak current of 0.1 µM 4-Cl-PIMT. (**D**) Effect of pH on the *E_p_* of 0.1 µM 4-Cl-PIMT. (**E**) The proposed reduction mechanism of 4-Cl-PIMT at the surface of SPCE/CNFs.

The effect of the type of supporting electrolytes on the voltammetric response of 0.05 and 0.1 µM 4-Cl-PIMT was checked using 0.1 M solutions of H_2_SO_4_, HNO_3_, CH_3_COOH, and acetate buffers with pH values of 3.5 ± 0.1, 4.0 ± 0.1, and 5.0 ± 0.1, respectively; the corresponding data are depicted in Figure 4A. It is clearly visible that the highest peak current of the reduction was obtained in acidic media. Figure 4B shows a comparison of the SWV curves recorded for 0.1 µM 4-Cl-PIMT in 0.1 M solutions of acetic (red), sulfuric (blue), and nitric (black) acids. Considering the obtained results, the nitric acid solution was found to be the most suitable for the determination of 4-Cl-PIMT. Moreover, the concentration of nitric acid from 0.01 to 0.125 M was evaluated (Figure 4C). The highest and best-shaped analytical signal of 4-Cl-PIMT was attained for the 0.025 M concentration of HNO_3_, so it was selected for subsequent experiments.

In addition, the linearity of peak potential (E_p_) of 4-Cl-PIMT vs. pH plot (Figure 4D) was obtained within the pH range of 1.0–5.0 (r = 0.9910). The equation slope is identical to the theoretical value of 0.059 V pH^−1^, indicating that the electrode process involves an equal number of protons and electrons. This is demonstrated in the reduction mechanism of our anticancer agent candidate (Figure 4E). It was found that the reduction process of 4-Cl-PIMT occurs at the surface of screen-printed carbon electrode modified with carbon nanofibers (SPCE/CNFs)—as a sensor—through an electron-gain mechanism, with the transfer of two electrons and two protons. The analyte possesses two azomethine moieties of the ketimine-type (C=N). The reduction of each of these azomethine groups (C3=N2, C8a=N1) implies the same number of protons and electrons. On the basis of previous findings [29,30,31,32], it was proposed that the C=N moiety located at positions 3 and 2 would be exclusively susceptible to the electrochemical reduction under these experimental conditions, leading to a protonated CH–NH group. This proposal may be supported by the proven susceptibility of this azomethine moiety to electrochemical reduction in structures of monocyclic, as well as fused, triazinones with single N–N bonds [29,30,31]. In addition, this may be supported by the experimentally observed reduction peak potential, the value of which is close to that of the same azomethine moiety of the ketimine-type undergoing regioselective electrochemical reduction in a structurally similar triazinone [32].

### 2.3. CV Studies

**Figure 5:**
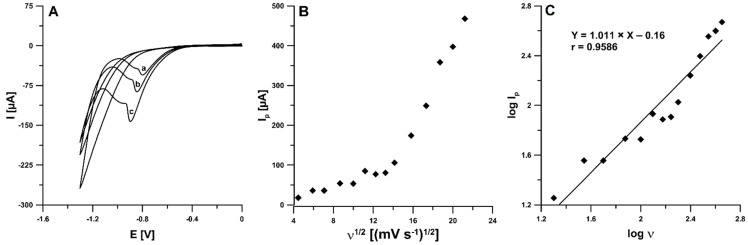
(**A**) Cyclic voltammograms of 10.0 µM 4-Cl-PIMT at ν of 50 (a), 100 (b), and 200 (c) mV s^−1^. (**B**) The dependence between I_p_ and ν^1/2^. (**C**) The dependence between logI_p_ and logν for ν from 20 to 450 mV s^−1^.

The electrochemical responses of 4-Cl-PIMT (10.0 µM) at the SPCE/CNFs in 0.025 M HNO_3_ were examined using cyclic voltammetry (CV). The CV curves were recorded for different values of scan rate (ν) from 20 to 450 mV s^−1^. Figure 5A shows the CV curves for the selected ν of 50, 100, and 200 mV s^−1^. In the potential range used, one irreversible cathode peak was visible at -0.88 V (ν = 100 mV s^−1^). The reduction peak potential shifted toward more negative values with the increase in scan rate, which confirmed that the analyte (4-Cl-PIMT) was irreversibly reduced. More information about the electrochemical behaviour of 4-Cl-PIMT at the SPCE/CNFs was obtained based on the measured values of the reduction peak current at various scan rates (20–450 mV s^−1^). The dependence between the intensity of the peak current (I_p_) and the square root of the scan rate (ν^1/2^) was plotted (Figure 5B), the non-linear course of which indicates that the 4-Cl-PIMT reduction process on the SPCE/CNF surface was adsorption-controlled. Furthermore, the relationship between the log of the peak current (logI_p_) and the log of the scan rate (logν) was plotted (Figure 5C). The slope of 1.011 in the plot of logI_p_ vs. logν indicates that this process was purely adsorption-controlled [33].

### 2.4. Effect of SWAdSV Parameters

**Figure 6:**
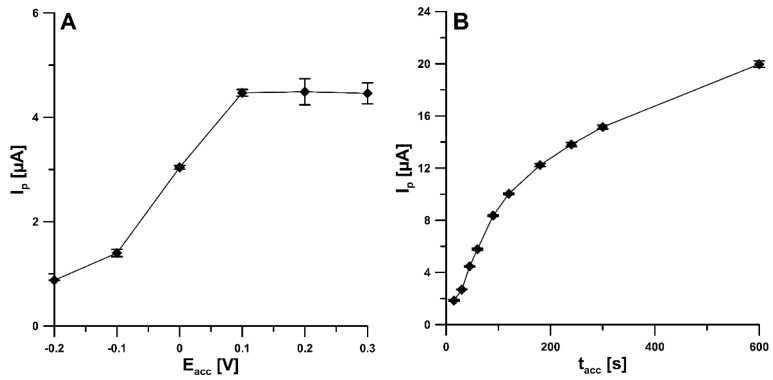
Influence of E_acc_ (**A**) and t_acc_ (**B**) on the peak current intensity of 20.0 nM 4-Cl-PIMT. The mean values of I_p_ are given with the standard deviation for *n* = 3.

Due to the confirmation of the adsorption of the 4-Cl-PIMT onto the surface of SPCE/CNFs by CV, we decided to evaluate the influence of parameters such as the accumulation potential (E_acc_) and time (t_acc_) on the peak current of this compound. The accumulation potential was varied over the range from −0.2 to 0.3 V, and t_acc_ was equal to 45 s. Figure 6A shows that the peak current increased when the accumulation potential was changed from −0.2 V towards more positive values and reached its maximum intensity at 0.1 V. Further increasing the potential value did not increase the analytical signal. For the selected value of E_acc_, the effect of accumulation time was investigated in the range of 15–600 s. As can be seen, in Figure 6B, the highest peak current was obtained for an accumulation time of 600 s; however, to make the analysis less time consuming, 120 s was chosen for further study. To further improve the detection limit, an accumulation time greater than 120 s is recommended.

**Figure 7:**
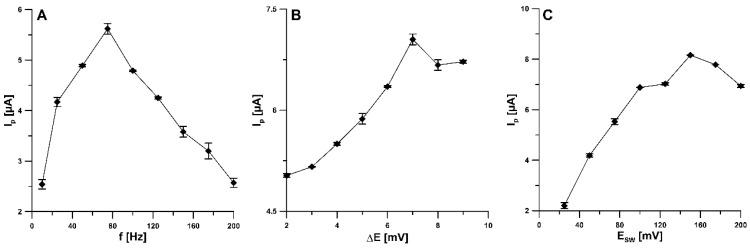
Effect of f (**A**), ΔE (**B**), and E_SW_ (**C**) on the analytical signal of 20 nM 4-Cl-PIMT. E_acc_ of 0.1 V and t_acc_ of 120 s. The mean values of I_p_ are given with the standard deviation for *n* = 3.

The influence of the square-wave frequency (f) on the analytical signal of the 20.0 nM 4-Cl-PIMT was studied in the range of 10–200 Hz (Figure 7A). For further research, we decided to choose an f value of 75 Hz. The next step was to select the appropriate step-potential (ΔE) value. For this purpose, the influence of this parameter was investigated from 2 to 9 mV (Figure 7B). An increase in the peak current was observed as the step potential increased to 7 mV and then decreased. Therefore, 7 mV was considered the optimal value. In addition, an influence of the square-wave amplitude (E_SW_) was checked over the range from 25 to 200 mV (Figure 7C, f of 75 Hz and ΔE of 7 mV). The highest 4-Cl-PIMT peak current was obtained for the E_SW_ value of 150 mV.

### 2.5. Analytical Performance

**Figure 8:**
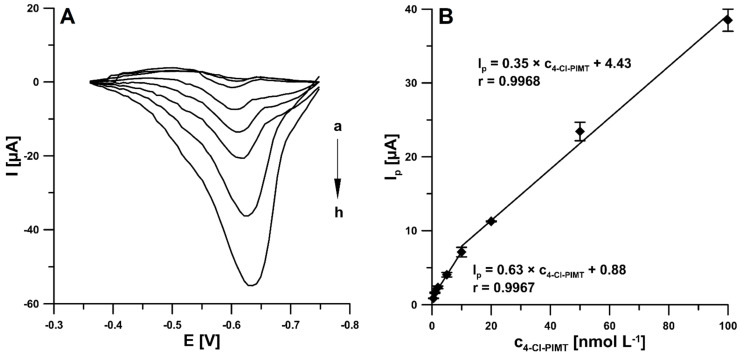
(**A**) Voltammograms obtained at the SPCE/CNFs in the 0.025 M solution of HNO_3_ containing increasing concentrations of 4-Cl-PIMT: (a) 0.5, (b) 1.0, (c) 2.0, (d) 5.0, (e) 10.0, (f) 20.0, (g) 50.0, and (h) 100.0 nM. (**B**) Calibration plot of 4-Cl-PIMT. SWAdSV parameters: E_acc_ of 0.1 V, t_acc_ of 120 s, f of 75 Hz, ΔE of 7 mV, and E_SW_ of 150 mV. The mean values of I_p_ are given with the standard deviation for *n* = 3.

Under the optimised experimental conditions presented above, the 4-Cl-PIMT was analysed on the SPCE/CNFs by SWAdSV. The reduction current responses were found to be proportional in the two linear ranges of 0.5–10 nM and 10–100 nM (Figure 8A,B). This is probably connected with the fact that at concentrations higher than 10 nM, saturation took place or the analyte transport mechanism was altered. The limits of detection (LOD) and quantification (LOQ) were calculated at 0.099 and 0.33 nM, respectively, using the following formulas: LOD = 3SDa/b and LOQ = 10SDa/b (SDa, standard deviation of intercept (*n* = 3); b, slope of calibration curve) [34].

The 4-Cl-PIMT analytical peak at the SPCE/CNFs displays very good repeatability, with an RSD of 2.2% (20.0 nM 4-Cl-PIMT, *n* = 10). To examine the reproducibility of SPCE/CNFs, three different sensors were applied for the analysis of 20.0 nM 4-Cl-PIMT. The RSD value of 5.1% (*n* = 9) confirmed the acceptable reproducibility of the SPCE/CNFs.

### 2.6. Selectivity

**Figure 9:**
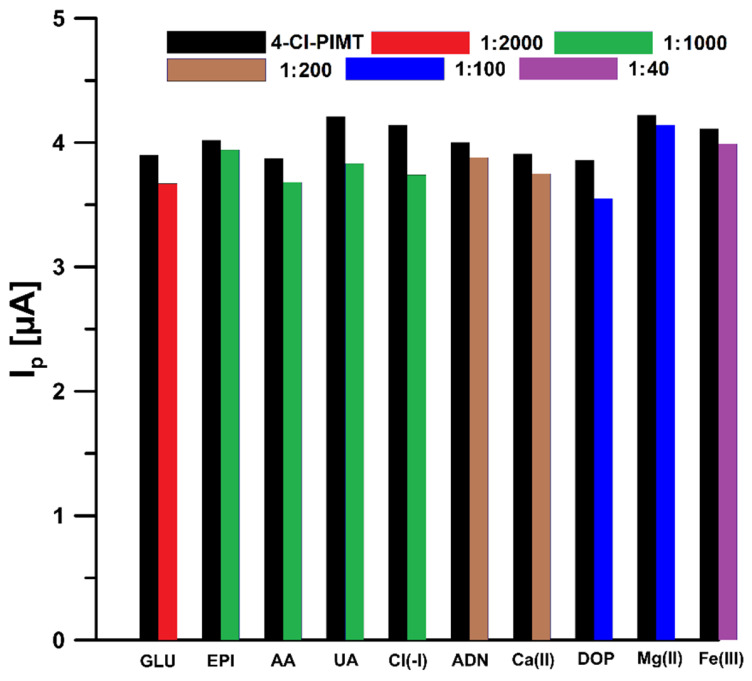
Histogram bars of the 5 nM 4-Cl-PIMT peak current in the presence of interferents. GLU—glucose, EPI—epinephrine, AA—ascorbic acid, UA—uric acid, ADN—adenine, DOP—dopamine.

**Figure 10:**
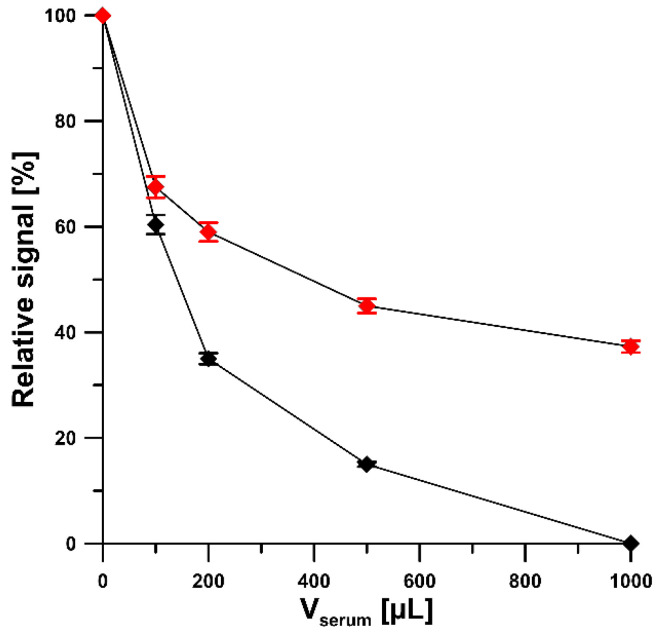
Effect of human serum on the 5 nM 4-Cl-PIMT peak current in the classic (black) and flow (red) systems.

We examined the effect of interferences that can potentially occur in biological fluids on the electrochemical response of 4-Cl-PIMT. The tolerance limit was defined as the concentration that gave an error of ≤10% in the assay of 5 nM 4-Cl-PIMT. It was observed that glucose (up to 2000-fold excess), epinephrine (up to 1000-fold excess), ascorbic acid (up to 1000-fold excess), uric acid (up to 1000-fold excess), Cl(-I) (up to 1000-fold excess), adenine (up to 200-fold excess), Ca(II) (up to 200-fold excess), dopamine (up to 100-fold excess), Mg(II) (up to 100-fold excess), and Fe(III) (up to 40-fold excess) had negligible effects on the assay of 4-Cl-PIMT (Figure 9). Considering that the developed procedure is intended to be used for the determination of 4-Cl-PIMT in biological samples, the influence of human serum on the analytical signal of 5 nM 4-Cl-PIMT was also investigated. Due to the significant influence of the serum matrix on the analyte peak current, we decided to use a flow system in the analysis of real samples in order to reduce the influence of interferents present therein. A comparison of the effect of serum on the voltammetric response of 5 nM 4-Cl-PIMT in the classical and flow systems is presented in Figure 10.

### 2.7. Serum Sample Analysis

In order to confirm the usefulness of the developed voltammetric procedure using SPCE/CNFs, 4-Cl-PIMT was determined in spiked human serum samples. As mentioned in Section 2.6, in order to minimise interference from the sample matrix, a flow system was used, in which, after the accumulation step, a supporting electrolyte solution was introduced to the flow cell, followed by recording of the voltammograms. This procedure was aimed at removing the sample solution from the near-electrode space in order to reduce the probability of reducing interferents at the stage of recording the analytical signal. Additionally, for the same purpose, we decided to reduce the accumulation time from 120 to 30 s. Table 1 summarises the results obtained in the flow electrochemical cell. The recovery values obtained by SWAdSV were 100.7 and 105.0%. The SWAdSV results were compared to those obtained by ultra-high-performance liquid chromatography coupled with electrospray ionization/single-quadrupole mass spectrometry (UHPLC-ESI-MS). As can be seen, the relative error values between the results obtained by SWAdSV and UHPLC-ESI-MS are satisfying, amounting to 2.18 and 9.48%, respectively.

## 3. Materials and Methods

### 3.1. The Investigated Electroactive Molecule

4-Cl-PIMT was chosen for the current electrochemical research and was freshly re-synthesised from 1-(4-chlorophenyl)-2-hydrazinylideneimidazolidine hydroiodide (as the starting nucleophilic building block) and α-oxophenylacetic acid (as the electrophilic annulation reagent), according to one of the original synthetic approaches described in [1]. The analyte had a fully defined molecular structure, as all its spectroscopic data (see Supplementary Material Table S1) were consistent with those previously reported [1]. This highly pure white organic compound had a sharp melting point (275–276 °C) after recrystallization from a binary solvent mixture of dichloromethane/methanol. Furthermore, when dissolved in buffer/acetonitrile and injected into the immobilised-artificial-membrane (IAM) column, the 4-Cl-PIMT revealed a symmetric peak of a Gaussian shape in its chromatogram (Figure S1 in the Supplementary Materials).

### 3.2. Apparatus

Voltammetric measurements were performed using a µAutolab analyser (Eco Chemie, Utrecht, The Netherlands) controlled by GPES 4.9 software in an electrochemical quartz cell with a commercially available screen-printed sensor (Metrohm-DropSens, Oviedo, Spain). The same analyser controlled by FRA 4.9 software was also used to record Nyquist plots in the electrochemical impedance spectroscopy (EIS) method. The three-electrode commercially available sensors consisted of a carbon working electrode unmodified or modified with carbon nanofibers or multiwalled carbon nanotubes, as well as a carbon auxiliary electrode and a silver pseudo-reference electrode (SPCE, ref. 110; SPCE/CNFs, ref. 110CNF and SPCE/MWCNTs, ref. 110CNT). The experiments on the flow system were carried out using a peristaltic pump-type MS-CA (Ismatec, Wertheim, Germany) and a commercially available methacrylate wall-jet flow cell (FLWCL, DropSens, Llanera, Spain). Chromatographic measurements were conducted using an Agilent Technologies 1200 Infinity ultra-high-performance liquid chromatography system consisting of an autosampler, degasser, binary pump, and column thermostat connected to an Agilent Technologies 6120 quadrupole mass spectrometer equipped with an electrospray ionization source (API-ESI) (Wilmington, DE, USA). Chromatographic separation was achieved on a Zorbax Eclipse Plus C18 rapid-resolution HT (2.1 × 50 mm, 1.8 µm) analytical column protected by a Zorbax Eclipse Plus-C18 narrow-bore guard column (2.1 × 12.5 mm, 5 µm), both purchased from Agilent Technologies.

### 3.3. Reagents and Solutions

The solutions of sulfuric acid, acetic acid, nitric acid, and acetate buffers of different pH were prepared from Sigma-Aldrich reagents. Merck (Darmstadt, Germany) standard solutions of Ca(II), Mg(II), Fe(II), and Cl(-I), as well as Sigma-Aldrich (Saint Louis, MO, USA) reagents (adenine, dopamine, epinephrine, glucose, uric acid, and ascorbic acid) were used in interference studies. For voltammetric measurements, a 1.0 mM solution of 4-Cl-PIMT was prepared in N,N-dimethylformamide (Sigma-Aldrich, Saint Louis, MO, USA). For UHPLC-ESI-MS analysis, acetonitrile (Merck, Darmstadt, Germany), formic acid (LC-MS, Sigma, Saint Louis, MO, USA), and trichloroacetic acid (TCA, Sigma-Aldrich, Saint Louis, MO, USA) were used. A stock solution of the analyte (1 g L^−1^) was prepared in dimethyl sulfoxide (DMSO, Merck, Darmstadt, Germany). Working solutions of the analyte were prepared in 0.1% (*v*/*v*) formic acid in acetonitrile. All solutions were prepared using ultra-purified water (>18 MΩ cm, Milli-Q system, Millipore, UK).

### 3.4. 4-Cl-PIMT SWAdSV Analysis

Voltammetric measurements of 4-Cl-PIMT were carried out in a classic electrochemical cell under optimised conditions in 0.025 M solution of HNO_3_. An accumulation potential (E_acc_) of 0.1 V was applied during stirring for 120 s (accumulation time, t_acc_). Voltammograms were recorded within the potential range from −0.2 to −1.0 V with a frequency (f) of 75 Hz, a step potential (ΔE) of 7 mV, and a square-wave amplitude (E*_SW_*) of 150 mV. The background curve was subtracted from each voltammogram. In the flow system during serum sample analysis, in the first step 0.025 M solution of HNO_3_ containing the spiked sample for 40 s was directed through the cell in order to accumulate the analyte on the surface of SPCE/CNFs. During this step, an accumulation potential of 0.1 V was used. Then, 0.025 M HNO_3_ was directed to the cell for 10 s in order to remove the sample solution, and voltammograms were recorded. The average values of I_p_ are shown with the standard deviation of *n* = 3.

### 3.5. 4-Cl-PIMT UHPLC-ESI-MS Analysis

Volumes of 0.1% (*v*/*v*) formic acid in water (A) and acetonitrile (B) were used as solvents for elution (flow rate of 0.3 mL min^−1^). The gradient used for the analysis was as follows: 15% B at 0 min, 70% B at 7–8 min, 15% B at 10 min (post run: 2 min). The injection volume and column temperature were 5 µL and 40 °C, respectively. Spectra were recorded in positive-ion mode, with a capillary voltage of 4000 V, nebuliser pressure of 45 psi, drying gas flow of 11 L min^−1^ at 350 °C, and fragmentor voltage of 140 V. Selected ion monitoring (SIM) was used to record the abundance of the [M + H]^+^ ion peak at *m*/*z* 325.1 (retention time: ~6.78 min)

### 3.6. Serum Sample Analysis

Normal human serum purchased from Merck (Darmstadt, Germany) was tested. Frozen human serum was thawed at room temperature. Then, 100 μL of the human serum sample was 100 times diluted in deionised water spiked with appropriate concentrations of the analyte, transferred to a centrifugal tube, mixed with 50 µL of 7.5% (*w*/*v*) TCA solution (Sigma-Aldrich, Saint Louis, MO, USA) in order to precipitate proteins, centrifuged at 4000× *g* for 10 min, and filtered through a 0.22 µm Millipore filter. The supernatant was analysed in triplicate by the optimised voltammetric procedure and UHPLC-ESI-MS method.

## 4. Conclusions

In the present studies, a screen-printed carbon electrode modified with carbon nanofibers (SPCE/CNFs) was proposed as the sensor in the first analytical procedure allowing for the selective and sensitive determination of the most promising anticancer drug candidate from a class of disubstituted fused triazinones, i.e., 4-Cl-PIMT. The increase in the analytical signal of the 4-Cl-PIMT at the SPCE/CNFs compared to an unmodified SPCE results from an increase in the active surface of the working electrode and a reduction in the charge-transfer resistance. The developed SWAdSV procedure is characterised by good sensitivity and selectivity. The calculated LOD and LOQ values were found to be 0.099 and 0.33 nM, respectively. The developed procedure using SPCE/CNFs was successfully used to determine the title analyte in human serum samples. The flow system minimised the effect of human serum matrix on the analyte signal. The SWAdSV results were compared to those obtained by the UHPLC-ESI-MS method, and the relative error values between the results obtained by both methods proved to be satisfying (2.18% and 9.48%). The 4-Cl-PIMT analytical peak at the SPCE/CNFs as a sensor displayed high reproducibility and repeatability.

## Figures and Tables

**Table 1:** Results of 4-Cl-PIMT determination in human serum samples.

4-Cl-PIMT Concentration [nM] ± SD (*n* = 3)					
Added	Found SWAdSV	Found UHPLC-ESI-MS	Recovery * SWAdSV [%]	Recovery ** UHPLC-ESI-MS [%]	Relative Error *** [%]
2.0 20.0	2.10 ± 0.072 20.13 ± 0.55	2.32 ± 0.34 19.7 ± 0.30	105.0 100.7	116.0 98.5	9.48 2.18

* Recovery [%] = (Found SWAdSV × 100)/Added; ** Recovery [%] = (Found UHPLC-ESI-MS × 100)/Added; *** Relative error [%] = ((ǀFound UHPLC-ESI-MS—Found SWAdSVǀ)/Found UHPLC-ESI-MS) × 100.

## Data Availability

The data presented in this study are available on request from the corresponding author. A sample of the investigated compound (4-Cl-PIMT) is available from the corresponding author.

## Supplementary Materials

The following supporting information can be downloaded at: https://www.mdpi.com/article/10.3390/ijms23052429/s1.
