# Supplementary material for: Application of a Screen-Printed Sensor Modified with Carbon Nanofibers for the Voltammetric Analysis of an Anticancer Disubstituted Fused Triazinone
Source: Int J Mol Sci. 2022 Feb 23;23(5):2429. doi: 10.3390/ijms23052429 (PMC8910141; doi:10.3390/ijms23052429)
Supplement: Supplementary file 1 [file ijms-23-02429-s001.zip › ijms-1611783-supplementary.pdf]

Table S1. Spectroscopic data for the 4-Cl-PIMT:.

^{1}H NMR ($CDCl_3$/TFA)	δ (ppm): 4.50 (t, 4H, imidazolidine CH_2–CH_2), 7.38-8.15 (m, 9H, aromatic–H)
IR (KBr)	ν (cm^{-1}): 3104, 3082, 3062, 2999 (aromatic C–H), 1687 (C=O), 1581, 1504, 1450 (aromatic skeleton), 1554 (C=N), 1257 (C–N), 1091 (aromatic C–Cl), 797 (1,4-disubstituted benzene ring), 750, 700 (monosubstituted benzene ring)
UV (MeOH)	λ_{max} (ε): 268 nm (14120); λ'_{max} (ε): 339 nm (9790)

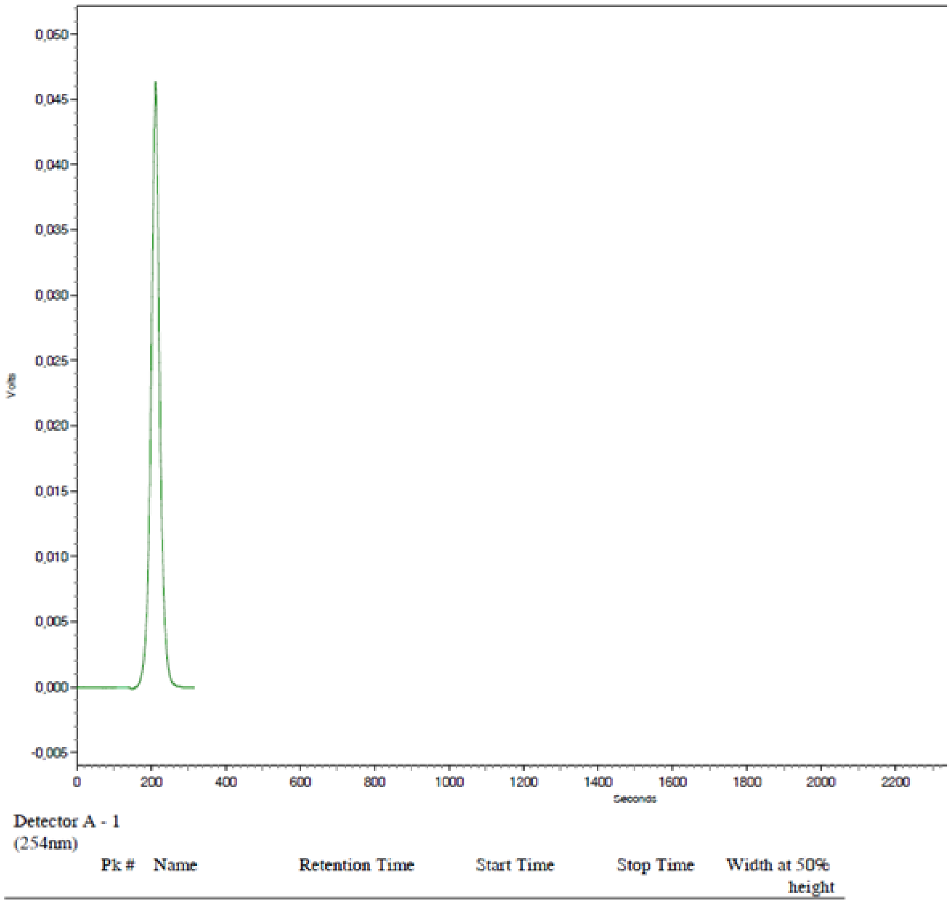

Figure S1. The peak for the 4-Cl-PIMT obtained on the IAM column (IAM.PC.DD2 100 × 4.6 mm i.d., 10 μm; Regis Chemicals Company, Morton Grove, Illinois, USA). Chromatographic system: buffer (Na_2HPO_4 / citric acid; pH = 7.4) / acetonitrile (6:4, v/v), temperature 25 °C.
